# Lipidated Analogs of the LL-37-Derived Peptide Fragment KR12—Structural Analysis, Surface-Active Properties and Antimicrobial Activity

**DOI:** 10.3390/ijms21030887

**Published:** 2020-01-30

**Authors:** Elżbieta Kamysz, Emilia Sikorska, Maciej Jaśkiewicz, Marta Bauer, Damian Neubauer, Sylwia Bartoszewska, Wioletta Barańska-Rybak, Wojciech Kamysz

**Affiliations:** 1 Laboratory of Chemistry of Biological Macromolecules, Department of Molecular Biotechnology, Faculty of Chemistry, University of Gdańsk, 80-308 Gdańsk, Poland; 2 Laboratory of Structural Studies of Biopolymers, Department of Organic Chemistry, Faculty of Chemistry, University of Gdańsk, 80-308 Gdańsk, Poland; emilia.sikorska@ug.edu.pl; 3 Department of Inorganic Chemistry, Faculty of Pharmacy, Medical University of Gdańsk, 80-416 Gdańsk, Poland; mj@gumed.edu.pl (M.J.); marta.bauer@gumed.edu.pl (M.B.); damian.neubauer@gumed.edu.pl (D.N.); sylwia.bartoszewska@gumed.edu.pl (S.B.); wojciech.kamysz@gumed.edu.pl (W.K.); 4 Department of Dermatology, Venereology and Allergology, Faculty of Medicine, Medical University of Gdańsk, 80-214 Gdańsk, Poland; wioletta.baranska-rybak@gumed.edu.pl

**Keywords:** ESKAPE pathogens, *Staphylococcus aureus*, KR12, LL-37, lipopeptide, critical aggregation concentration, CD spectroscopy, NMR, biofilm, cytotoxicity

## Abstract

An increasing number of multidrug-resistant pathogens is a serious problem of modern medicine and new antibiotics are highly demanded. In this study, different n-alkyl acids (C_2_-C_14_) and aromatic acids (benzoic and *trans*-cinnamic) were conjugated to the *N*-terminus of KR12 amide. The effect of this modification on antimicrobial activity (ESKAPE bacteria and biofilm of *Staphylococcus aureus*) and cytotoxicity (human red blood cells and HaCaT cell line) was examined. The effect of lipophilic modifications on helicity was studied by CD spectroscopy, whereas peptide self-assembly was studied by surface tension measurements and NMR spectroscopy. As shown, conjugation of the KR12-NH_2_ peptide with C_4_-C_14_ fatty acid chains enhanced the antimicrobial activity with an optimum demonstrated by C_8_-KR12-NH_2_ (MIC 1–4 μg/mL against ESKAPE strains; MBEC of *S. aureus* 4–16 μg/mL). Correlation between antimicrobial activity and self-assembly behavior of C_14_-KR12-NH_2_ and C_8_-KR12-NH_2_ has shown that the former self-assembled into larger aggregated structures, which reduced its antimicrobial activity. In conclusion, *N*-terminal modification can enhance antimicrobial activity of KR12-NH_2_; however, at the same time, the cytotoxicity increases. It seems that the selectivity against pathogens over human cells can be achieved through conjugation of peptide *N*-terminus with appropriate n-alkyl fatty and aromatic acids.

## 1. Introduction

The occurrence of multidrug-resistant (MDR) bacterial strains faces many difficulties in the therapy of some infections due to prolonged treatment and frequent relapses. An increasing number of MDR pathogens is mainly associated with persistent and abused use of antibiotics and just those strains are mostly associated with hospital flora. The ESKAPE pathogens (*Enterococcus faecium, Staphylococcus aureus*, *Klebsiella pneumoniae*, *Acinetobacter baumannii*, *Pseudomonas aeruginosa* and *Enterobacter* spp.) are bacterial species responsible for most of nosocomial infections [1]. Moreover, recent epidemiological data have shown that the therapy of infections caused by those bacteria is also associated with the highest risk of mortality [2]. According to the latest reports of World Health Organization (WHO), all of the ESKAPE pathogens are listed in the group of bacteria for which new antibiotics are highly demanded [3]. It should be noted that one of the species in the high priority group are methicillin-resistant *S. aureus* (MRSA), which are prevalent species in environment making them the major source of hospital-acquired infections (HAIs) [4]. It has been estimated that almost 44% of all HAIs are caused by those bacteria, with indication of being responsible for over 20% of excessive mortality [5,6]. The therapy of infections caused by MRSA is even more challenging as these strains produce a number of mechanisms allowing them to invade into the organisms, including avoidance of opsonization by antibodies and complement system, disruption of chemotaxis and lysis of neutrophils. Because of their ability to survive inside leukocytes, the infections tend to move into a chronic stage and recur after recovering. Furthermore, the therapy often needs prolonged hospitalization and commonly tends to be ineffective. An additional complication of the therapy is the ability of bacteria to form biofilms—an organized three-dimensional structure characterized by enhanced resistance to antibiotics [7]. It has been estimated that approximately 80% of chronic and recurrent infections are associated with the biofilm occurrence [8]. Low effectiveness of the current approaches to the therapy of HAIs together with accompanying side-effects adversely affect the patient’s health. A multitude of antibiotics often fail to be effective in the treatment because of MDR strains. Therapeutic difficulties accompanying the majority of infections escalates the need to search for new effective drugs. Antimicrobial peptides (AMPs) are a promising class of antimicrobial compounds which have a chance to fight resistant pathogens owing to their rapid membrane-targeting bactericidal mode of action and the predicted low propensity for development of resistance [9,10,11]. One of the AMPs is a linear, cationic, α-helical and amphipathic peptide LL-37 (LLGDFFRKSKEKIGKEFKRIVQRIKDFLRNLVPRTES), the member of the human cathelicidin family [12,13,14]. This peptide is released from its precursor, hCAP-18, through proteolytic processing by proteinase 3, a serine proteinase secreted from neutrophils [14]. Interestingly, the hCAP-18 found in seminal plasma can also be hydrolyzed by vaginal gastricsin. As a result, instead of LL-37 another peptide (ALL-38) can be generated. Although this compound contains additional alanine at the *N*-terminus, exhibits comparable antimicrobial activity [15]. Furthermore, LL-37 is found in variety of cells, tissues and body fluids such as leukocytes, bone marrow, milk, salivary glands, skin, respiratory tract, epithelium cells and leukocytes within the digestive tract, urinary tract as well as squamous epithelium of the mouth and tongue [12,13,16,17]. This compound exhibits a broad spectrum of antibacterial activity against both planktonic cells and biofilms of Gram-positive and Gram-negative bacteria, which promotes it as a candidate for a new antibiotic [18,19]. However, LL-37 is a relatively long peptide, which makes it to be expensive for manufacturing. Thus, the search for a novel, shorter analogs of LL-37 is desired. Some fragments of LL-37 have been evaluated to identify improved antimicrobial derivatives (for instance KR12, FK-13, GF17, 17BIPHE2) [20,21,22,23,24]. Both LL-37 and its shorter active analogs adopt a helical structure in the presence of membrane lipids [23,24,25]. The shortest α-helical fragment of LL-37 with documented antimicrobial activity is KR12 amide (KRIVQRIKDFLR-NH_2_) [20,21,26]. This peptide, a truncated form of LL-37, shares two common features of antimicrobial peptides: a positive net charge and an amphipathic structure, which determine their antimicrobial activity.

In this article, we report the synthesis of a series of lipopeptides derivatized with variable length fatty acids or aromatic acids covalently attached to the *N*-terminus of KR12-NH_2_, and their antimicrobial activity against planktonic cells of ESKAPE bacteria. The fatty acid tail was introduced to KR12-NH_2_, because in the literature it was found that addition a fatty acid residue to AMPs may improve effectiveness of peptides as antimicrobial agents by enhancing their ability to form either secondary structures or oligomerize upon interacting with bacterial membranes [27,28,29]. We also demonstrate the activity of the tested peptides against biofilm formed by reference strains of *S. aureus* (including *MRSA*), because one of the obstacles complicating therapy of staphylococcal infections is the growth of biofilm. Relationships between antimicrobial activity and hemolytic activity as well as cytotoxicity of the peptides were also determined. The effect of *N*-terminal modifications on helicity of the KR12-NH_2_ peptide was studied by CD spectroscopy. The ability of the selected lipopeptides to spontaneous self-assembly in solution was evaluated with surface tension measurements and NMR spectroscopy.

## 2. Results and Discussion

This section describes and discusses results MS and RP-HPLC analyses of peptides (2.1), evaluation of their antimicrobial activity against planktonic *S. aureus* and ESKAPE strains and biofilm of *S. aureus* reference strains (2.2), as well as studies on hemolysis (2.3) and cytotoxicity (2.4). Moreover, CD spectroscopy (2.5), critical aggregation concentration (CAC) and NMR spectroscopy (2.6) were included to learn how *N*-terminal modification affects secondary-structure and peptide self-assembly. As found, the activity of the studied peptides was determined by many concurrent parameters, including hydrophobicity, conformation or tendency to self-assembly.

### 2.1. Peptide Synthesis and Purification

Peptides **I**-**X** were synthesized by solid-phase method using Fmoc chemistry. Their purity was higher than 95% as shown by analytical reversed-phase high-performance liquid chromatography (RP-HPLC). The electrospray ionization mass spectrometry (ESI MS) in positive ion mode confirmed identity of the purified peptides. Physicochemical characteristics of the peptides are shown in Table 1.

**Figure 1:**
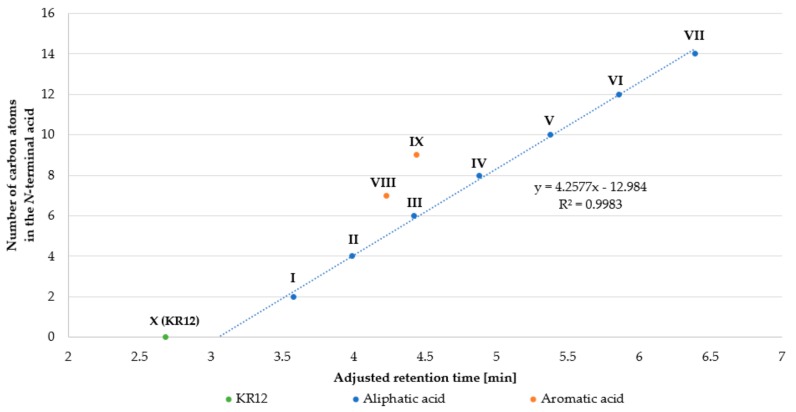
The number of carbon atoms in the *N*-terminal acid residue *versus* adjusted retention time.

Conjugation of KR12-NH_2_ *N*-terminal amino group with aliphatic or aromatic acids result in compounds with a reduced net charge (+4 vs. +5) and enhanced hydrophobicity, as shown by RP-HPLC. Results of this evaluation are presented in Table 1. Moreover, the number of carbon atoms in the *N*-terminal acid was plotted against the adjusted retention time (Figure 1).

Peptides with *N*-terminal aliphatic acid to generate a homologous series differ in the number of carbon atoms (methylene groups). It can be seen that retention time (hydrophobicity) of peptides **I**–**VII** increased proportionally to the number of carbon atoms (linear regression, *R*^2^ = 0.9983). Retention time of KR12-NH_2_ (+5) is shifted to lower values than those predicted using regression equation of **I**–**VII** (calculated 3.05 vs. measured 2.68 min), mainly due to its higher net charge (+5). Analogs with an aromatic acid at the *N*-terminus did not follow this trend. The calculated retention times of analogs with identical number of carbon atoms in aliphatic acids as compared to those with aromatic **VIII** (C_7_) and **IX** (C_9_) acids were distinctly higher (more hydrophobic) than the experimentally determined ones (**VIII**: 4.23 vs. 4.69 min calc.; **IX**: 4.44 vs. 5.16 min calc.). This phenomenon is the result of different carbon hybridization. In aromatic acids, carbon atoms are sp^2^ hybridized and in the aliphatic ones they are sp^3^ hybridized (excluding carbon atom of carboxylic group), which influences polarity, shape and planarity (aromatic ring) of aromatic compounds, and both can affect retention time [30,31,32].

### 2.2. Antimicrobial Assay

**Figure 2:**
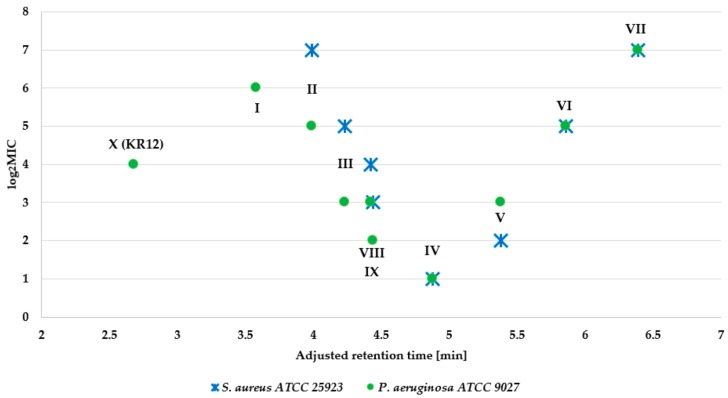
Antimicrobial activity of KR12 amide and its analogs (log_2_MIC) against *S. aureus* ATCC 25923 and *P. aeruginosa* ATCC 9027 *versus* adjusted retention time.

In our preliminary research, we tested LL-37 and KR12-NH_2_ (**X**) against a reference strain of *S. aureus* ATCC 25923. Minimal inhibitory concentrations (MICs) of *S. aureus* strain were 256 μg/mL for peptide KR12-NH_2_ and >512 μg/mL for LL-37 in analysis performed in the Mueller-Hinton medium. MICs for *S. aureus* strain cultivated in 1% Bacto Peptone medium were 64 μg/mL for peptide KR12-NH_2_ and >512 for LL-37. We also tested antimicrobial activity of LL-37 and KR12-NH_2_ against clinical strains of *S. aureus* acquired from the skin and nose and it strongly depended on the bacterial strains of *S. aureus* (MICs values ranged between 1 and >512 μg/mL) [33]. Because antistaphylococcal activities of KR12-NH_2_ and LL-37 were comparable, we decided to introduce a lipophilic residue to peptide KR12-NH_2_ (**X**). Peptide **X** and its nine analogs (**I**–**IX**) were tested against selected reference strains of ESKAPE bacteria (Table 2—*E. faecium*, *K. pneumoniae*, *A. baumannii*, *P. aeruginosa*, *K. aerogenes*; Table 3—several reference strains of *S. aureus* including *MRSA* ATCC 33591) and staphylococcal biofilm (Table 4). The antimicrobial activity of the synthesized peptides was dependent on the number of carbon atoms in the *N*-acyl substituent. A high activity against planktonic forms of the bacteria and the staphylococcal biofilm was found for peptides **III**–**V** and **IX** (Table 2, Table 3 and Table 4). The most effective was the analog of KR12-NH_2_ modified in the *N*-terminal part of the molecule with octanoic acid residue (C_8_-KR12-NH_2_, peptide **IV**) for which the minimal inhibitory concentration (MIC) values ranged between 1 and 4 μg/mL (Table 2 and Table 3), while the minimal biofilm eradication concentrations (MBECs) of *S. aureus* strains were four-fold higher than the MIC values and ranged between 4 and 16 μg/mL. Generally, the conjugation of the KR12-NH_2_ with both longer and shorter hydrocarbon acyl chains than that of C_8_ resulted in a decrease in antimicrobial activity. The next active compound was analog KR12-NH_2_ modified with *trans*-cinnamic acid residue (peptide **IX**). For this particular compound, the MIC values ranged between 1 and 8 μg/mL (Table 2 and Table 3). The MBEC values were four-fold higher than those of MIC values and ranged between 4 and 32 μg/mL. As a rule, modification of the KR12 amide with fatty acid residues (C_4_-C_14_) intensified antimicrobial potency against the tested bacteria. An exception was found only for analog modified with C_4_ (**II**), which was inactive against *A. baumannii*. In general, antimicrobial activity of the analogs depended on their hydrophobicity (*N*-terminal acid). The relation between antimicrobial activity of the peptides against Gram-positive (*S. aureus* ATCC 25923) and Gram-negative (*P. aeruginosa* ATCC 9027) strains and their hydrophobicity (adjusted retention time) is presented on the Figure 2.

Positive charge of the peptide is essential for its antimicrobial activity due to interactions with negatively charged pathogen cells. A gradual reduction of positive charge usually results in decrease or loss of antimicrobial activity [34,35]. However, as observed, there was no simple correlation between charge and activity. In case of the studied analogs the *N*-terminal modification reduced net charge from +5 to +4. Despite the reduction of total positive charge, most of the analogs displayed improved antimicrobial activity when compared to KR12-NH_2_. This finding emphasizes that *N*-terminal modification also modifies other structural parameters of the peptide that are crucial for activity. The antimicrobial activity of lipopeptides depended on the length of the acyl substituent, which is compatible with earlier reports [27,36,37]. However, in the literature, different fatty acids have been suggested as the optimum modification to provide enhanced antimicrobial activity. For instance, Laverty et al. conjugated a tetrapeptide amide H-Orn-Orn-Trp-Trp-NH_2_ with saturated fatty acids (C_6_-C_16_) and demonstrated that *N*-acyl substituents of 12–14 carbon atoms in length exhibited the strongest antimicrobial and antibiofilm activities [36]. As a result, the hydrophobicity of the *N*-acyl substituent was pointed as a key determinant of antimicrobial activity for the peptides [36]. Albada et al. also studied the influence of lipidation (C_2_-C_14_) on antimicrobial potency of short active unnatural AMPs. In this case, the highest activities against a broad spectrum of pathogens were found for compounds modified with C_8_ and C_10_ residues. The authors suggested that the lowered activity of peptides lipidated with C_12_ and C_14_ could be associated with their poor solubility in media used for microbiological assays [37]. In our study, the optimum modification was found for lipidation with octanoic acid (C_8_).

### 2.3. Hemolysis Assay

**Figure 3:**
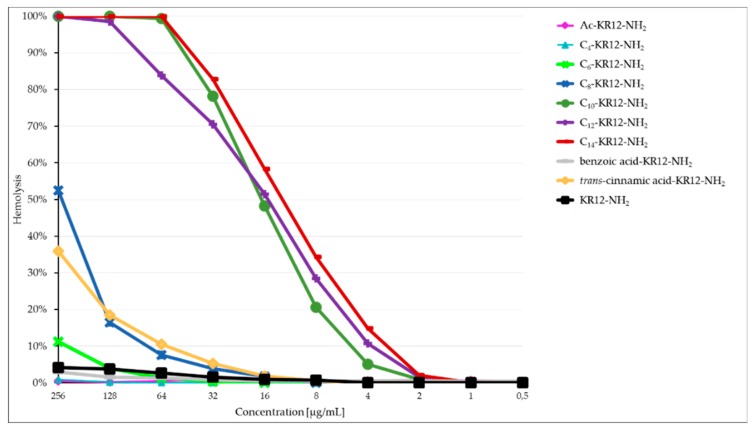
Percentage of hemolysis of erythrocytes *versus* peptide concentration.

The hemolytic activity of peptides **I**–**X** was assessed for human red blood cells (hRBCs) to verify their toxicity (Figure 3 and Table 5). Our results indicate that hemolysis of the erythrocytes depended on the number of carbon atoms of the conjugated acid. For longer hydrocarbon acyl chains (C_2_ to C_14_), the hemolytic activity increased. For instance, for peptides **I** (C_2_—ethanoic acid) and **II** (C_4_—butyric acid) the hemolysis of hRBCs was not detected within the studied concentration range (0.5–256 μg/mL). Analogs **III** and **IV** (with hexanoic acid – C_6_ and octanoic acid – C_8_) exhibited a higher hemolytic activity, but it was still below their MIC and MBEC values. The most effective compound against both planktonic cells and the biofilm of *S. aureus* was peptide **IV,** which caused 5% hemolysis of hRBCs at a concentration of 64 μg/mL. Peptide **IV** had a high selectivity index amounting to almost 28 (Table 5). In the case of peptides, **V**–**VII** containing hydrocarbon acyl chains from C_10_ to C_14_ the hemolytic activity was comparable and exceeded the values of MIC and MBEC. For these compounds, hemoglobin release was found, beginning from 2–4 μg/mL. Lysis of hRBCs for those peptides was noticed in the range of 64–128 μg/mL. As a result, an analog with a tetradecanoic acid (**VII**) turned out to be the most toxic one. Noteworthy is the fact that conjugation of KR12-NH_2_ with aromatic carboxylic acids, such as benzoic (C_7_) and *trans*-cinnamic (C_9_) acids led to analogs **VIII** and **IX** that were less hemolytic than analogs **III** (C_6_) and **IV** (C_8_). However, selectivity index of peptide **IX** was lower than that of peptide **IV** (Table 5). Peptide **VIII** did not cause hemolysis of hRBCs (>5%) over the whole concentration range. SI value for this peptide was not calculated because minimal hemolytic concentration (MHC) exceeded 256 μg/mL. It should be emphasized that peptide KR12-NH_2_ (**X**), which was used as a base for all modifications, did not cause any significant hemolysis (>5%) over the whole concentration range (0.5–256 μg/mL).

### 2.4. MTT Assay

All the tested peptides exhibited cytotoxicity against human keratinocytes cell line (HaCaT). A 65-fold difference in half maximal inhibitory concentration (IC_50_) values was observed between peptides displaying the highest and lowest degrees of cytotoxicity (Table 5). Only for peptides **III–V**, **VIII** and **IX**, the IC_50_ values were higher than the mean MIC ones (GM) for *S. aureus*. Moreover, the highest toxicity was found for peptide **VII** (C_14_), but its activity against *S. aureus* was relatively poor. On the other hand, the least toxic was peptide **I**. Therefore, for this compound the antimicrobial activity was determined only against *E. faecium* and *P. aeruginosa* (16 and 64 μg/mL, respectively). Selectivity indices IC_50_/GM against reference strains of *S. aureus* were low. The most selective were analogs modified with aromatic acid residues **VIII** (C_7_) and **IX** (C_9_) with selectivity indices of 2.20 and 2.50, respectively. Several articles on the safety-profile of KR-12 analogs have already been published, indicating high antimicrobial activity and low cytotoxicity of the aforementioned analogs. For instance, in the study of Jacob et al. a series of KR12 analogs were designed and synthesized in order to optimize the α-helical structure (KR12-a1 to a7) [21]. As a result, all of the analogs showed insignificant cytotoxicity against macrophages of RAW264.7 cell line and anti-inflammatory activity. Moreover, on the basis of these results, Kim et al. conducted a research on d-amino acid substituted analogs of KR-12-a5 (KRIVKLILKWLR-NH_2_) which appeared to be non-toxic against macrophages (RAW264.7) and fibroblasts (NIH-3T3) at whole concentration range [38]. It is worth noticing that Rajasekaran et al. conducted research on alanine scan of FK-13 peptide (FKRIVQRIKDFLR-NH_2_), which is also considered to be an antimicrobial region of LL-37 [22]. In this study, the cytotoxicity was determined against both RAW264.7 and HaCaT cell lines. As a result, for majority of peptides the viability of test cells at MIC concentrations were not significantly affected. Furthermore, in the last two cases, the cell selectivity (therapeutic index) was determined in the relation to hemolysis. Respectively, in the study of Kim et al. the most selective was compound with 6-dl, with selectivity of 61.2, while in the article of Rajasekaran et al. the most selective was FK13-a4 with a selectivity equal to 138.4 [22,38]. However, the obtained results should not be compared as in one assay the MHC that caused 10% of hemolysis was taken into calculation while in another considered concentration was HC50 (50% hemolysis).

**Figure 4:**
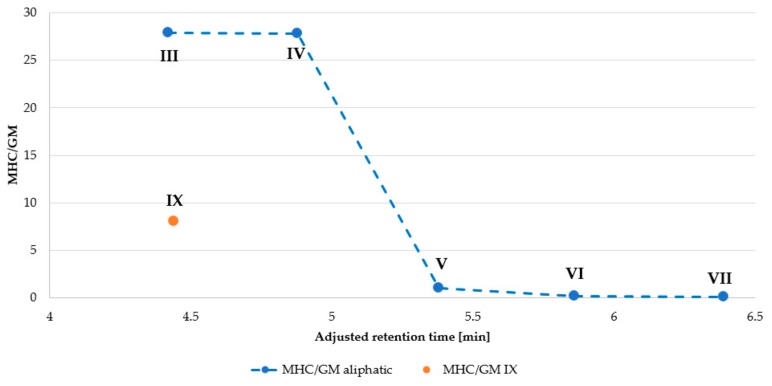
Selectivity for *S. aureus* over erythrocytes *versus* adjusted retention time.

**Figure 5:**
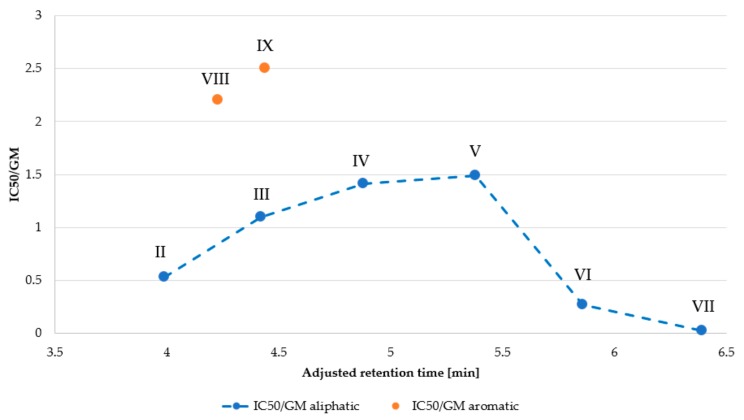
Selectivity for *S. aureus* over human cells (HaCaT) *versus* adjusted retention time.

Peptide net charge and hydrophobicity can affect biological activity, including antimicrobial potency and cytotoxicity as well as also selectivity. Data in Table 1 and Table 5 were used to find a relationship between selectivity and hydrophobicity of KR12-NH_2_ analogs (Figure 4 and Figure 5). As seen, longer acyl chain and higher hydrophobicity influenced hemolytic activity to a greater extent than antimicrobial activity, resulting in reduced selectivity indices MHC/GM (Figure 4). Similar tendency was observed in case of selectivity indices IC_50_/GM; however, the differences between the peptides are not so spectacular (Figure 5). Interestingly, KR12-NH_2_ modified with aromatic acids (analogs **VIII** and **IX**) had highest selectivity indicies (IC_50_/GM). It has been shown that conjugation of the *N*-terminal amino group of the cationic peptide (Orn-Orn-Trp-Trp-NH_2_) with cinnamic acid (and its derivatives) lead to compounds with promising antimicrobial activity against Gram-positive bacteria (*S. aureus*) and low cytotoxicity (HaCaT cell line) and hemolytic activity [40].

### 2.5. Conformational Studies

**Figure 6:**
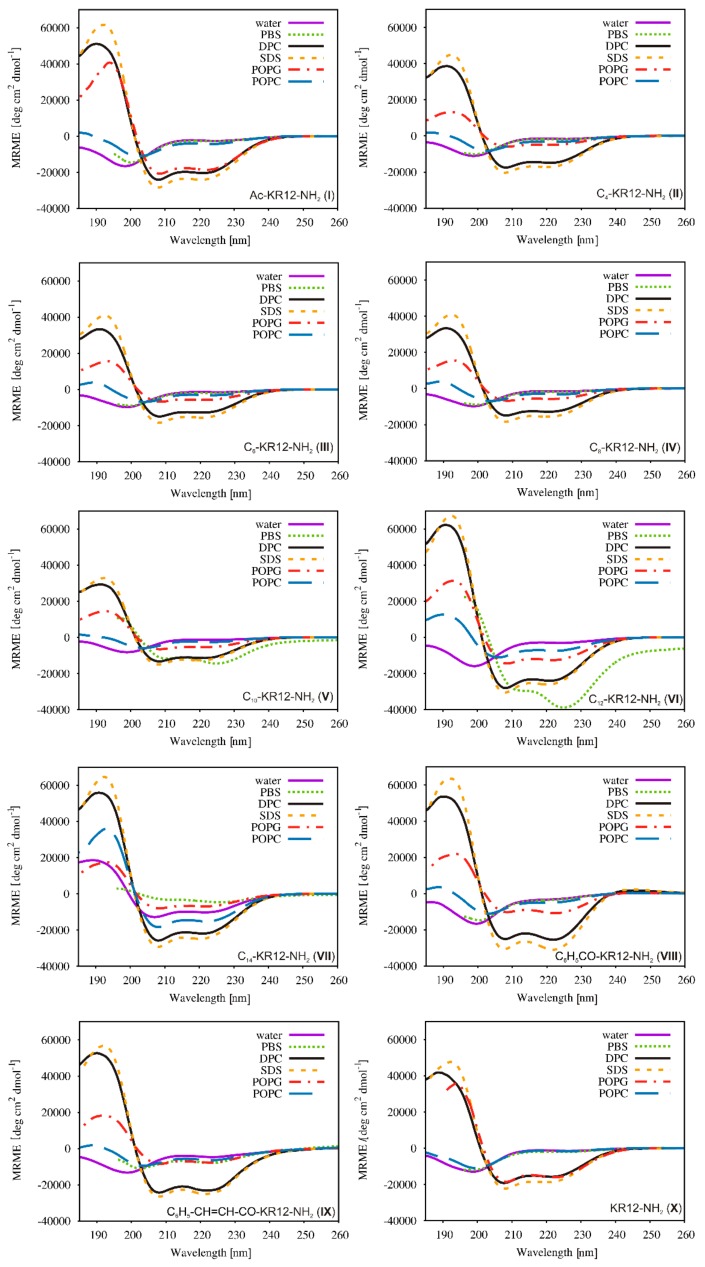
Far-UV CD spectra of the peptides.

The CD spectra (Figure 6) revealed the peptides to be generally devoid of a stable conformation in water and phosphate buffered saline (PBS) solutions, and only addition of membrane-mimicking surfactants such as sodium dodecyl sulfate (SDS) and dodecylphosphocholine (DPC) as well as liposomes such as 1-palmitoyl-2-oleoyl-sn-glycero-3-phosphocholine (POPC) and 1-palmitoyl-2-oleoyl-sn-glycero-3-phosphoglycerol (POPG) induced an α-helical structure, but lipopeptides C_10_-KR12-NH_2_ (**V**), C_12_-KR12-NH_2_ (**VI**) and C_14_-KR12-NH_2_ (**VII**) clearly deviated from this trend. As seen, the CD spectra of compounds **V**, **VI** and **VII** in PBS, as well as of analog **VII** in water, displayed typical features of peptides with an α-helix folding with two well-defined minimums at 208 and 222 nm. A straightforward explanation of this fact is a self-assembly of the lipopeptides, which seems to be a sufficient factor to provide hydrophobic environment stabilizing the helical conformation. In the case of C_14_-KR12-NH_2_ (**VII**), hydrophobic interactions between the tetradecanoic acyl chains were sufficient to overcome the electrostatic and steric repulsion between the peptide residues in both non-buffered and buffered aqueous solutions. In turn, with C_10_-KR12-NH_2_ (**V**) and C_12_-KR12-NH_2_ (**VI**), only an increase in the solution ionic strength resulted in an effective screening of the electrostatic peptide repulsion leading to peptide’s self-assembly. Interestingly, a Θ222/Θ208 ratio greater than 1 noticed in PBS indicated a coiled-coil formation and was a further proof for self-assembly. A similar tendency has previously been observed for conjugates of magainin with lipophilic acids [41].

As seen in Figure 6, there were only insignificant differences in the CD spectra of the peptides in SDS and DPC micelles. In both detergents, a high helical content was found for all the peptides studied (45–86%, Table 6). In turn, POPG and POPC liposomes had a different effect on conformation of the peptides. In general, the presence of POPC liposomes, a model of eukaryotic membranes, induced an increase in helicity with elongation of the attached acyl chain, which correlated well with a rise in hemolytic activity. The C_14_-KR12-NH_2_ (**VII**) analog with the highest helical percentage in POPC liposomes exhibited also the highest cytotoxicity against human keratinocytes. On the other hand, analogs Ac-KR12-NH_2_ (**I**) and C_8_-KR12-NH_2_ (**IV**) displayed the same helical fraction (16%) but extremely different cytotoxicity, because the former was found to be the least cytotoxic, whereas the latter one of the most cytotoxic peptides. This indicated no linear correlation between helicity and toxicity of the peptides studied. In turn, the CD spectra in POPG liposomes, representing a negatively charged bacterial membrane, showed that modifications of KR12-NH_2_ with acyl chains longer than that of C_4_ as well as aromatic substituents (C_7_ and C_9_) reduced the helical fraction, but without any clear correlation with antimicrobial activity. This result was not surprising at all because numerous studies to date have shown different relationships between helicity and antimicrobial activity. In particular, Shai and Oren have demonstrated that reducing helicity by incorporating d-amino acids decreased hemolytic activity but did not affect most of the potent antimicrobial activity of the diastereomeric analogs as compared to that of the parent peptides [42,43]. Comparable results have been reported for Temporin l analogs [44]. In turn, a study on enantiomers of Pleurocidin [45] has shown all the d-amino acid-containing peptides exhibited a decreased antibacterial activity and a dramatically decreased hemolytic activity as that of compared to l-amino acid-containing counterpart despite a higher percentage of helical structure. All this suggests that conformation of the peptides is not the only factor affecting biological activity.

### 2.6. Self-Assembly Studies

**Figure 7:**
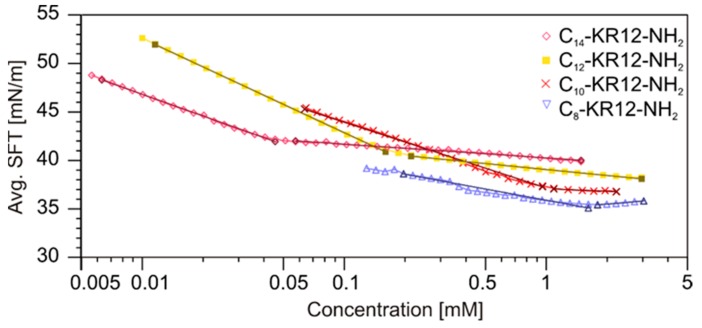
Relationship between the surface tension and peptide concentration.

The critical aggregation concentrations (CACs) were determined for the analogs with *N*-terminal fatty acids C_8_-C_14_ (Figure 7). The surface tension measurements were carried out in pure water, because the peptides aggregated in PBS solution. This is probably the consequence of self-assembly at much lower CAC values according to the rule that an increase in ionic strength of the solution decreases the CAC value of ionic surfactants [46,47,48].

**Figure 8:**
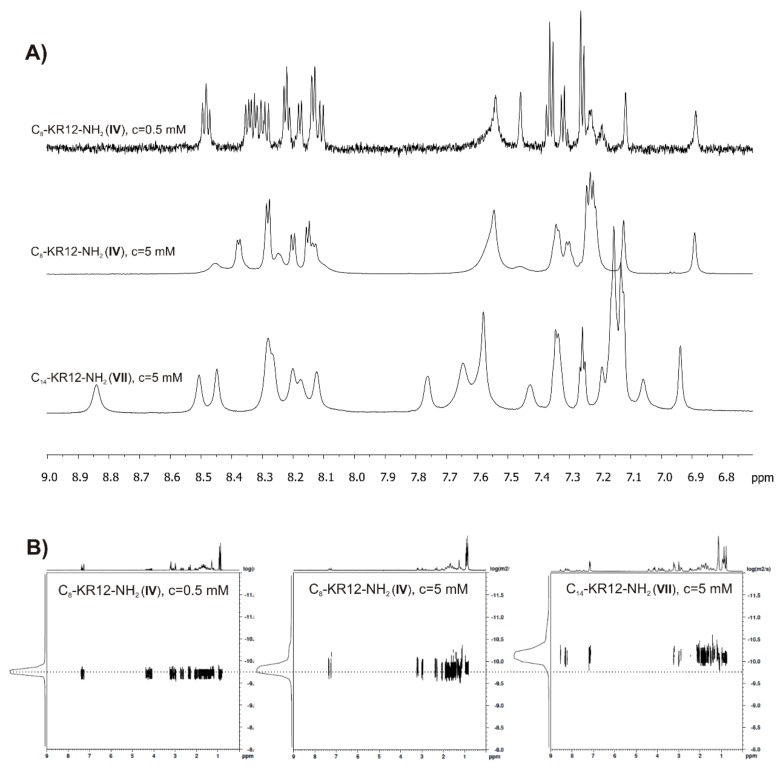
^1^H NMR (**A**) and DOSY (**B**) spectra for C_8_-KR12-NH_2_ and C_14_-KR12-NH_2_ recorded at concentrations below and/or above critical aggregation concentration (CAC).

As expected, the longer was the lipophilic acyl chain, the more effective self-assembly became, owing to an increase in intermolecular hydrophobic interactions. The CAC values decreased with increasing alkyl chain length following the order: 1.46 mM (2475 µg/mL), 1.05 mM (1807 µg/mL), 0.17 mM (297 µg/mL) and 0.042 mM (74 µg/mL) for **IV**, **V**, **VI** and **VII**, respectively. In the ^1^H NMR spectra, the self-assembly induced broadening of the resonance lines (Figure 8A), which is related to a decrease in system’s tumbling rate and shortening of the T_2_ relaxation times. Interestingly, the NMR spectra of C_8_-KR12-NH_2_ (**IV**) and C_14_-KR12-NH_2_ (**VII**), both recorded at a concentration above CAC, differed from each other despite identical peptide sequences, reflecting different conformations of the peptides. In the latter case, the amide proton resonances were spread out over a wider range of chemical shifts as compared to that of the former, this being characteristic of formation of helical structure [49]. The translation diffusion coefficient (*D*_tr_) determined at a concentration higher than CAC of C_14_-KR12-NH_2_ (**VII**) and the corresponding hydrodynamic radius (R_H_), derived from the Stokes-Einstein’s equation were 7.76 × 10^−11^ m^2^/s and 32 Å, respectively. Due to the low CAC value, it was difficult to measure the self-diffusion coefficient for the monomer of peptide **VII**. Hence, the length of a single molecule was established to be ~35 Å assuming a tetradecanoic acyl tail and the peptide moiety to exist in full-extended and helical conformations, respectively. This value corresponded well with R_H_ extracted from the translation diffusion coefficient. Therefore, we concluded that C_14_-KR12-NH_2_ self-assembled into micelles. In the case of C_8_-KR12-NH_2_ (**IV**), the translation diffusion coefficients, *D*_tr_, extracted from the NMR experiments at concentrations three-fold lower and three-fold higher than CAC were 1.95 ×∙10^−10^ and 1.74 × 10^−10^ m^2^/s, respectively, and corresponded to the *D*_tr, oligomer_/*D*_tr, monomer_ ratio of roughly 0.89. Based on the previous study, the *D*_tr, oligomer_/D_tr, monomer_ ratio of ~0.8 is related to dimer formation by assuming that both the monomer, and the dimer adopt compact (spherical) structures. With the peptides of elongated shapes, this ratio may increase [50,51]. For comparison, in the case of common surfactants, SDS and l,2-diheptanoyl-sn-grycero-3-phosphocholine (DHPC), as well as other antimicrobial lipopeptides, which self-assemble into spherical micelles, the *D*_tr, micelle_/*D*_tr, monomer_ ratio is lower than 0.5 [52,53,54]. Taking all this into account, we speculate that C_8_-KR12-NH_2_ self-assembled into dimers and the oligomerization over the tested concentration range does not favor helix formation.

## 3. Materials and Methods

### 3.1. Peptide Synthesis

The peptides (Table 1) were synthesized by solid-phase method using Fmoc chemistry on a resin modified by a Rink amide linker with a loading of 1.0 mmol/g (Orpegen Peptide Chemicals GmbH, Heidelberg, Germany) [55,56]. *N*^α^-Fmoc-protected amino acids and the coupling reagents were obtained from Iris Biotech GmbH (Marktredwitz, Germany). The following amino acids side-chain-protecting groups were used: Trt (for Gln), OtBu (Asp), Boc (Lys), Pbf (Arg). Peptide synthesis was carried out manually. Single deprotection of the Fmoc group was performed in a 20% (*v*/*v*) piperidine (Iris Biotech GmbH, Marktredwitz, Germany) solution in *N*,*N*-dimethylformamide (DMF) for 15 min. Acylation with a protected amino acid was conducted in a dichloromethane (DCM)/DMF (Merck, Poland) solution with coupling agents for 1.5 h using a 3-fold molar excess of *N*,*N*′-diisopropylcarbodiimide (DIC; Peptideweb, Zblewo, Poland) and OxymaPure (Iris Biotech GmbH, Marktredwitz, Germany). Every step was preceded by rinsing the resin and running the chloranil test. Coupling reactions of lipophilic residues (fatty acids, aromatic acids) were performed by the same method as that used for protected amino acids. After the synthesis, the peptide resins were dried under vacuum. The peptides were cleaved from the resin using a mixture of trifluoroacetic acid (TFA; Apollo Scientific, Denton, UK), triisopropylsilane (TIS; Sigma-Aldrich, St. Louise, MO, USA), and water (95:2.5:2.5 *v*/*v*/*v*). The cleaved peptides were precipitated with cold diethyl ether and lyophilized. All peptides were purified using the RP-HPLC on a Knauer system controlled by an LPchrom data system (Lipopharm.pl, Zblewo, Poland) with a Knauer Kromasil C8 column (8 × 250 mm, 100Å pore size, 5 μm particle size). The eluates were fractionated and analyzed by analytical RP-HPLC. The purity of the peptides was determined on a Varian ProStar HPLC system controlled by a Galaxie Chromatography Data System with Phenomenex Gemini-NX C18 column (4.6 × 150 mm, 110 Å pore size, 5 μm particle size). The solvent systems used were: 0.1% aqueous TFA (A) and 0.1% TFA in acetonitrile (ACN) (B). UV detection at 214 nm was used, and the peptides were eluted with a linear gradient 10–100% B in A over 10 min at 25 ± 0.1 °C. The mobile phase flow rate was 2.0 mL/min. The ESI MS (Waters Alliance e2695 system with Acquity QDA detector, Waters, Milford, MA, USA) was used to identify the masses of the obtained peptides.

### 3.2. Antimicrobial Assays

#### 3.2.1. Microbial Strains and Antimicrobial Assay

Examination of antimicrobial activity of the test compounds was conducted on reference strains of bacteria assigned to ESKAPE group of pathogens: *Enterococcus faecium* ATCC 700221, *Klebsiella pneumoniae* ATCC 700603, *Acinetobacter baumannii* ATCC BAA-1605, *Pseudomonas aeruginosa* ATCC 9027, *Klebsiella aerogenes* ATCC 13048 (formerly *Enterobacter aerogenes*) and reference strains of *Staphylococcus aureus*, namely: *S. aureus* ATCC 25923, *S. aureus* ATCC 6538, *S. aureus* ATCC 33591 (MRSA), *S. aureus* ATCC 9144 and *S. aureus* ATCC 12598. All the strains were stored at −80 °C in Roti^®^-Store cryo vials (Carl Roth GmbH, Karlsruhe, Germany) and before the tests were transferred into fresh Mueller-Hinton Medium (BioMaxima, Lublin, Poland) and incubated for 24 h at 37 °C. Subsequently, each bacterial inoculum was seeded on Mueller-Hinton Agar plates (BioMaxima) and incubated again for 24 h. The cultures prepared in this way were used in further antimicrobial assays and prepared as described above. The MIC values were determined by the broth microdilution method according to Clinical and Laboratory Standards Institute Protocol [57]. For this purpose, initial inoculums of bacteria (0.5 × 10^5^ colony forming unit (CFU)/mL) in Mueller–Hinton Broth were exposed to the ranging concentrations of compounds (0.5–256 µg/mL) and incubated for 18 h at 37 °C. The experiments were conducted on 96-well microtiter plates, with a final volume of 100 µL. Cell densities were adjusted spectrophotometrically (Multiskan™ GO Microplate Spectrophotometer, Thermo Scientific) at 600 nm. The MICs were taken as the lowest drug concentration at which a visible growth of microorganisms was inhibited [58]. All experiments were conducted in triplicate and included positive (growth) and negative (sterility) controls.

#### 3.2.2. Activity Against Staphylococcal Biofilm

The MBECs values were determined according to the method reported previously [59,60]. Briefly, 24-h cultures of *S. aureus*, namely: *S. aureus* ATCC 25923, *S. aureus* ATCC 6538, *S. aureus* ATCC 33591 (MRSA), *S. aureus* ATCC 9144 and *S. aureus* ATCC 12598 were diluted to a concentration of 0.5 × 10^7^ CFU/mL and added to the test wells of polystyrene microdilution flat-bottom plates. After 24-h of incubation at 37 °C, the wells were rinsed three times with PBS to remove non-adherent cells. Subsequently, 100 μL of the test compounds over a concentration range 0.5–256 µg/mL were added to each well and incubated again for 24 h at 37 °C. After this period, 20 μL of the resazurin (7-hydroxy-3H-phenoxazin-3-one-10-oxide, 4 mg/mL) solution was added to each well. After 1 h of incubation, the MBECs were read out. The determined values were recorded as the lowest concentration at which the reduction of resazurin (from blue to pink) was lower or equal to 10 ±  0.5% as compared to the positive (100%) and negative (0%) controls. All experiments were performed in triplicate.

### 3.3. The Hemolysis Assay

The assay was conducted according to the procedure described previously by Avrahami and Shai [28]. Briefly, the fresh human RBCs with ethylenediaminetetraacetic acid (EDTA) as anticoagulant were rinsed three times with PBS by centrifugation at 800× *g* for 10 min and resuspended in PBS. Serial dilution of peptides (0.5–256 µg/mL) was conducted in PBS on 96-well plates. Then the stock RBCs solution was added up to a final volume of 100 µL with a 4% concentration of erythrocytes (v/v). The control wells for 0 and 100% hemolysis were also prepared. They consisted of RBCs suspended in PBS and 1% of Triton-X 100, respectively. Then, the plates were incubated for 60 min at 37 °C and centrifuged at 800× *g* for 10 min at 4 °C (Sorvall ST 16R Centrifuge, Thermo Scientific). After centrifugation, the supernatant was carefully transferred to new microtiter plates and the release of hemoglobin was monitored by measurement of absorbance at 540 nm (Multiskan™ GO Microplate Spectrophotometer). Percentage of hemolysis was calculated based on wells with 100% hemolysis.

### 3.4. MTT Assay

The cytotoxicity of test compounds (IC_50_) was evaluated for human keratinocytes (HaCaT, ATCC) using classic MTT assay on 96-well plates [61]. In this assay, a colorimetric determination of the cell metabolic activity was carried out. Specifically, the color intensity reflects the number of live cells that can be measured spectrophotometrically. Briefly, the cell line was cultured in a Dulbecco’s modified Eagle Medium (Sigma-Aldrich) supplemented with 10% fetal bovine serum (v/v), 100 units/mL of penicillin, 100 μg/mL of streptomycin, and 2 mM l-glutamine and was kept at 37 °C in a humidified 5% CO_2_ incubator. A day after plating 500 cells per well, a series of concentrations (0.5–500 μg/mL) of the test compounds were added. Dimethyl sulfoxide (DMSO) was used as a control in cells at a final concentration of 1.0% (*v*/*v*), which was related to the maximal concentration of the solvent compounds used in the experiment. After 24 h of incubation with test compounds, a medium containing 1 mg/mL of MTT was added up to a final concentration of 0.5 mg/mL and subsequently incubated at 37 °C for 4 h. Then, the medium was aspirated and the formazan product was solubilized with DMSO. The background absorbance at 630 nm was subtracted from that at 570 nm for each well (Epoch, BioTek Instruments, Winooski, VT, USA). Six replicates were conducted for each concentration. All experiments were repeated at least twice and the resulting IC_50_ values were calculated with a GraFit 7 software (v. 7.0, Erithacus, Berkley, CA, USA).

### 3.5. CD Measurements

Circular dichroism studies were performed in water, 10 mM PBS buffer (pH 7.4), 20 mM SDS micelles, 20 mM DPC micelles, and 1.3 mM LUVs POPG and POPC liposomes. Large unilamellar vesicles (LUVs) were prepared according to the previously described procedure [62]. The CD spectra were recorded on a JASCO J-815 spectropolarimeter at 25 °C in the 185–260 nm range. The peptide concentration was 0.15 mg/mL. Every spectrum was scanned three times to amplify the signal-to-noise ratio. The spectra were plotted as a function of the mean residue molar ellipticity (MRME, degree cm^2^dmol^−1^) vs. wavelength (nm). Deconvolution of the CD spectra were carried out using CDPro software with CONTINILL algorithm and SMP56 database set [63].

### 3.6. Surface Tension Measurements

Surface tension measurements were performed to determine CAC of selected lipidated KR12-NH_2_ analogs. The measurements were carried out using a Wilhelmy plate method on a K100 tensiometer equipped with two micro-dispensers (Krüss GmbH, Hamburg, Germany). The average value of the surface tension for every concentration was obtained on the basis of 10 measurements. The standard deviations did not exceed 0.1 mN/m. The CAC was determined by plotting the surface tension against the logarithm of compound concentration and was found as the intersection of two lines that best fit through the pre- and post-CAC data.

### 3.7. NMR Measurements

The NMR spectra were acquired on a Bruker Avance III 700 MHz spectrometer running Topspin 3.2 software in D_2_O and H_2_O:D_2_O (9:1 *v*/*v*) solution at 298 K. The ^1^H NMR spectra with excitation sculpting water suppression were recorded with 16 k data points in F2 dimension. The translation diffusion coefficients (*D*_tr_) were measured by the standard Bruker pulse program (stebpgp1s19) with WATERGATE solvent suppression, 4k data points in the F2 dimension, 32 data points (gradient strengths) in the F1 dimension and with 2 s relaxation delay. The diffusion time (∆) and the maximum duration of gradient distance (δ) were 200 ms and 4 ms in all experiments, respectively. The spectra were processed and analyzed using Topspin 3.2 (BrukerBiospins, Rheinstetten, Germany)).

## 4. Conclusions

The modification of KR12 amide (**X**) with a lipophilic residue in the *N*-terminal part of the molecule has been found to be an effective way to fortify its antimicrobial activity. For each of the synthesized lipopeptides, the activity against *S. aureus* as well as against bacteria of the ESKAPE group depended on the number of carbon atoms in the substituent. For example, the analog of KR12-NH_2_ (**IV**) containing octanoic acid residue (C_8_) exhibited the highest potency against all organisms tested in planktonic form (MIC 1–4 μg/mL). Moreover, it was able to eradicate biofilms of *S. aureus* strains at relatively low concentrations (MBEC 4–16 μg/mL). Furthermore, this peptide was characterized by low toxicity against hRBCs (MHC 64 μg/mL). For HaCaT, the IC_50_ value was 3.23 μg/mL, but the highest SI values were found for peptides **III** and **IV** (MHC/GM amounting to almost 28) and peptide **IX** with IC_50_/GM ratio of 2.50.

As has previously been argued, fatty acid conjugation enhances the peptide-membrane interactions [64]. On the other hand, it can either induce or enhance ability to self-assemble in solution, which in turn can perturb the water-membrane partition equilibrium by protecting hydrocarbon chains from water phase, thereby reducing the possibility of peptide membrane insertion. However, aggregation can also increase selectivity of membrane-active anticancer and antimicrobial peptides by reducing effective peptide hydrophobicity and thus affinity towards membranes composed of neutral lipids, such as the outer leaflet of healthy eukaryotic cell membranes [65]. In the case of the peptides studied, an increase in the length of the attached alkyl chain enhanced propensity for self-assembly, promoted formation of larger aggregates and decreased antimicrobial activity, but not cytotoxicity of KR12-NH_2_ analogs. Interestingly, self-assembly induced also α-helix formation in analogs with C_10_-C_14_ lipophilic residues. The remaining peptides underwent a conformational switch typical for most antimicrobial peptides only in the presence of surfactants or lipids mimicking membrane environment. No correlation was found between helicity and activity of the peptides, which shows that the antimicrobial activity is the result of many factors. Those affecting activity include conformation, hydrophobicity, hydrophobic moment, charge and its distribution, size of the hydrophobic/hydrophilic domain or aggregation state in solution [35,65]. Conjugation of KR12-NH_2_ peptide with lipophilic acids affected all of them to clearly demonstrate the complexity of lipopeptide-membrane interactions with multiple interconnected phenomena contributing in the final activity.

Analog KR12-NH_2_ (**IV**), containing octanoic acid, has a strong potential to eliminate both planktonic cells of ESKAPE pathogens and the staphylococcal biofilm, as demonstrated in this study. After characterizing its proteolytic stability, this compound might be a useful peptide template for developing novel antimicrobial agents. We do not exclude the possibility of changes in the peptide sequence, because both LL-37 and its fragments can be degraded by proteases [66]. The literature describes LL-37 derivatives that displayed antistaphylococcal activity in vitro but also maintained their activity in the presence of physiological salts and human serum (analogs FK-13-a1 and FK-13-a7) and were active in vivo and/or ex vivo (17BIPHE2, SAAP-148) [22,24,67,68]. A supplementary examination of improvement of peptide **IV** selectivity index and its ability to prevent the biofilm formation should also be considered. In addition, the promising antimicrobial activity and low toxicity of peptide **IX** modified with *trans*-cinnamic acid residue is noteworthy, supporting further studies on improving selectivity index and potential application in staphylococcal infections.

Importantly, lipopeptides are already used in the therapy of bacterial infections. Daptomycin is applied in the treatment of systemic bacterial infections. Moreover, polymyxin B is administered parenterally in patients with bacteremia and urinary-tract infections. Unfortunately, the major disadvantage of polymyxin B treatment is its relatively high nephrotoxicity and neurotoxicity [69,70]. On the other hand, daptomycin therapy is associated with dose-dependent myopathy [71]. Lipoglycopeptides are another class of drugs available on the market. Dalbavancin is used in patients with acute bacterial skin and skin structure infections (ABSSSI). This drug is considered to be safe and well-tolerated in the treatment of ABSSSI [72]. Telavancin is another FDA approved lipoglycopeptide for treatment of complicated skin and skin structure infections (cSSSI). Both dalbavancin and telavancin disrupt membrane integrity and cell-wall synthesis [73,74]. Conjugation of a peptide with a fatty acid can increase its stability in serum, tissues and organs. It has been shown that lipidated peptides bind to serum albumin. Moreover, chain length plays pivotal role in peptide stability [41,75,76,77,78]. Presumably, conjugation of KR12-NH_2_ with a fatty acid at its *N*-terminus may lead to increased enzymatic stability. The most active and selective peptides in this study may be useful peptide templates for novel antimicrobial agents. Further studies should estimate peptides proteolytic stability, activity in animal infection models and the influence of the position of the lipophilic moiety within KR12-NH_2_ on both antimicrobial activity and toxicity. As is known from literature, changing the location of the fatty moiety from the *N*-terminus of the molecule to its *C*-terminus can lead to a decrease in hemolytic activity of the molecule while not adversely affecting its antibacterial activity [37].

## Figures and Tables

**Table 1:** Characteristics of the peptides.

Peptide	Name	Net Charge	HPLC *t*’_R_ (min)	Average Mass (Da)	MS Analysis		
					*z*	*m/z* calc.	*m/z* found
I	Ac-KR12-NH_2_ (C_2_-KR12-NH_2_)	+4	3.58	1612.96	2	807.49	807.29
					3	538.66	538.69
					4	404.25	404.47
II	C_4_-KR12-NH_2_	+4	3.99	1641.02	2	821.52	821.38
					3	548.01	547.73
					4	411.26	411.36
III	C_6_-KR12-NH_2_	+4	4.42	1669.07	2	835.54	835.16
					3	557.36	556.84
					4	418.28	418.35
IV	C_8_-KR12-NH_2_	+4	4.88	1697.12	2	849.57	849.46
					3	566.71	566.65
					4	425.29	425.31
					5	340.43	340.56
V	C_10_-KR12-NH_2_	+4	5.38	1725.18	2	863.60	863.43
					3	576.07	575.86
					4	432.30	432.20
VI	C_12_-KR12-NH_2_	+4	5.86	1753.23	2	877.62	877.03
					3	585.42	585.04
					4	439.32	439.18
VII	C_14_-KR12-NH_2_	+4	6.39	1781.28	2	891.65	891.42
					3	594.77	594.59
					4	446.33	446.04
VIII	Benzoic acid-KR12-NH_2_	+4	4.23	1675.03	2	838.52	838.14
					3	559.35	559.00
					4	419.77	419.76
IX	*trans*-Cinnamic acid-KR12-NH_2_	+4	4.44	1701.07	2	851.54	851.34
					3	568.03	567.58
					4	426.28	425.85
X	KR12-NH_2_ (KRIVQRIKDFLR-NH_2_)	+5	2.68	1570.93	2	786.47	786.23
					3	524.65	524.69
					4	393.74	393.94
					5	315.19	315.18

*z*—ion charge, *m*/*z*—mass to charge ratio; adjusted retention time (*t*’_R_) is an analyte’s retention time (*t*_R_) minus the elution time of an unretained peak (*t*_m_).

**Table 2:** The minimal inhibitory concentration (MIC) values (µg/mL) of the peptides against reference strains of ESKAPE pathogens.

Peptide	*E. faecium* ATCC 700221	*K. pneumoniae* ATCC 700603	*A. baumannii* ATCC BAA-1605	*P. aeruginosa* ATCC 9027	*K. aerogenes* ATCC 13048
**I**	16	>256	>256	64	>256
**II**	4	128	>256	32	128
**III**	2	16	16	8	16
**IV**	1	2	2	2	2
**V**	2	16	8	8	16
**VI**	4	32	16	32	32
**VII**	8	64	32	128	128
**VIII**	1	16	16	8	16
**IX**	1	4	4	4	4
**X**	8	>256	256	16	>256

**Table 3:** The MIC values (µg/mL) of the test peptides against reference strains of *S. aureus.*

Peptide	*S. aureus* ATCC 25923	*S. aureus* ATCC 6538	*S. aureus* ATCC 33591	*S. aureus* ATCC 9144	*S. aureus* ATCC 12598
**I**	>256	256	>256	>256	>256
**II**	128	32	128	64	64
**III**	16	4	16	8	8
**IV**	2	2	2	2	4
**V**	4	4	4	4	4
**VI**	32	32	16	32	32
**VII**	128	64	16	64	128
**VIII**	32	8	32	16	16
**IX**	8	2	4	4	4
**X**	>256	256	>256	>256	>256

**Table 4:** The MBEC values (µg/mL) of the test peptides against reference strains of *S. aureus.*

Peptide	*S. aureus* ATCC 25923	*S. aureus* ATCC 6538	*S. aureus* ATCC 33591	*S. aureus* ATCC 9144	*S. aureus* ATCC 12598
**I**	>256	>256	>256	>256	>256
**II**	256	128	128	128	128
**III**	32	16	16	16	16
**IV**	8	16	4	4	4
**V**	32	32	32	16	8
**VI**	256	256	128	64	32
**VII**	256	256	256	128	64
**VIII**	64	32	32	16	16
**IX**	32	8	16	8	4
**X**	>256	>256	>256	>256	>256

**Table 5:** MHC, IC_50_, GM and selectivity indices (SI) of peptides determined for reference strains of *S. aureus.*

Peptide	MHC ^1^ (µg/mL)	IC_50_ (µg/mL)	GM ^2^ (μg/mL)	Selectivity Index (SI) ^3^	
				MHC/GM	IC_50_/GM
**I**	>256.00	84.20	>256.00	NA	NA
**II**	> 256.00	38.73	73.52	NA	0.53
**III**	256.00	10.13	9.19	27.86	1.10
**IV**	64.00	3.23	2.30	27.83	1.41
**V**	4.00	5.97	4.00	1.00	1.49
**VI**	4.00	7.60	27.86	0.14	0.27
**VII**	4.00	1.29	64.00	0.06	0.02
**VIII**	>256.00	40.50	18.38	NA	2.20
**IX**	32.00	10.00	4.00	8.00	2.50
**X**	>256.00	74.60	>256.00	NA	NA

^1^ MHC is the minimal hemolytic concentration that caused 5% hemolysis of human red blood cells. ^2^ The geometric mean (GM) of the MIC values against *S. aureus* was calculated. ^3^ SI is the ratio of MHC/IC_50_ to GM. More selective compounds are characterized by the highest values of SI [39]. NA: not applicable; SI values were not calculated for compounds with MHC and/or GM values higher than 256 µg/mL.

**Table 6:** Helical content determined based on CD spectra.

Peptide	Helical Content %					
	Water	PBS	DPC	SDS	POPG	POPC
**I**	8	8	72	82	69	16
**II**	7	6	69	71	67	9
**III**	7	7	65	67	24	16
**IV**	6	6	53	62	27	16
**V**	6	51	45	54	26	15
**VI**	8	74	81	86	55	32
**VII**	35	17	77	84	29	63
**VIII**	8	5	73	82	41	15
**IX**	7	19	72	77	30	18
**X**	7	7	66	70	66	7

## Abbreviations

AMPs	antimicrobial peptides
Ac	acetyl group
Boc	*tert*-butyloxycarbonyl
C_4_	butanoic acid residue
C_6_	hexanoic acid residue
C_8_	octanoic acid residue
C_10_	decanoic acid residue
C_12_	dodecanoic acid residue
C_14_	tetradecanoic acid residue
CAC	critical aggregation concentration
CD	circular dichroism
CFU	colony forming unit
DCM	dichloromethane
DHPC	l,2-diheptanoyl-sn-glycero-3-phosphocholine
DIC	*N*,*N*′-diisopropylcarbodiimide
DMF	*N*,*N*-dimethylformamide
DMSO	dimethyl sulfoxide
DOSY	Diffusion-Ordered Spectroscopy
DPC	dodecylphosphocholine
EDTA	ethylenediaminetetraacetic acid
ESI MS	electrospray ionization mass spectrometry
ESKAPE bacteria	*Enterococcus faecium, Klebsiella pneumoniae, Acinetobacter baumannii, Pseudomonas aeruginosa, Klebsiella aerogenes, Staphylococcus aureus*
Fmoc	9-fluorenylmethoxycarbonyl
GM	geometric mean
HAIs	hospital-acquired infections
hRBCs	human red blood cells
IC_50_	half maximal inhibitory concentration
MBEC	minimal biofilm eradication concentration
MDR	multidrug-resistant
MHC	minimal hemolytic concentration
MIC	minimal inhibitory concentration
MRSA	methicillin-resistant *S. aureus*
MTT	3-(4,5-dimethylthiazol-2-yl)-2,5-diphenyltetrazolium bromide
Pbf	2,2,4,6,7-pentamethyl-dihydrobenzofuran-5-sulfonyl residue
PBS	phosphate buffered saline
POPC	1-palmitoyl-2-oleoyl-sn-glycero-3-phosphocholine
POPG	1-palmitoyl-2-oleoyl-sn-glycero-3-phosphoglycerol
RP-HPLC	reversed-phase high-performance liquid chromatography
SDS	sodium dodecyl sulfate
SI	selectivity index
SPPS	solid-phase peptide synthesis
TFA	trifluoroacetic acid
TIS	triisopropylsilane
WHO	World Health Organization
